# Prevalence of undiagnosed diabetes mellitus and its associated factors in urban Burkina Faso

**DOI:** 10.4102/jphia.v15i1.497

**Published:** 2024-09-16

**Authors:** Solo Traoré, Désiré L. Dahourou, Boyo C. Paré, Yempabou Sagna, Daniel Zemba, Douonibo P. Somé, Nomwindé C.J. Ouédraogo, Kalo R. Millogo, Lassina Séré, Toussaint Rouamba, Hervé Tiéno, Oumar Guira

**Affiliations:** 1 Department of Medicine and Medical Specialties, Ziniaré Regional Hospital, Plateau Central Healthcare Region, Ziniaré, Burkina Faso; 2 Department of Biomedical and Public Health, Research Institute of Health Sciences, Ouagadougou, Burkina Faso; 3 Department of Public Health, Training and Research Unit in Health Sciences, Joseph Ki-Zerbo University, Ouagadougou, Burkina Faso; 4 Higher Institute of Health Sciences, Nazi Boni University, Bobo-Dioulasso, Burkina Faso; 5 Department of Internal Medicine, Sourô Sanou University Hospital, Bobo-Dioulasso, Burkina Faso; 6 Department of Medicine and Medical Specialties, Tenkodogo Regional Hospital, Tenkodogo, Burkina Faso; 7 Department of Internal Medicine, Yalgado Ouédraogo University Hospital, Ouagadougou, Burkina Faso; 8 Department of Medicine and Medical Specialties, Fada N’Gourma Regional Hospital, Fada N’Gourma, Burkina Faso; 9 Department Internal Medicine, Tengandogo University Hospital, Ouagadougou, Burkina Faso; 10 Clinical Research Unit of Nanoro, Institute for Research in Health Sciences, Nanoro, Burkina Faso; 11 Training and Research Unit in Health Sciences, Joseph Ki-Zerbo University, Ouagadougou, Burkina Faso; 12 Department of Internal Medicine, Bogodogo University Hospital, Ouagadougou, Burkina Faso

**Keywords:** diabetes mellitus, prevalence, associated factors, urban environment, Burkina Faso

## Abstract

**Background:**

Community screening could be an effective strategy for identifying people with undiagnosed type 2 diabetes mellitus (T2DM) in low-income countries.

**Aim:**

This study aimed to estimate the prevalence of undiagnosed T2DM and its risk factors.

**Setting:**

This study was conducted in Ouagadougou, the capital of Burkina Faso.

**Methods:**

This was a cross-sectional study, including consenting population (≥ 18 years). Data were collected from 11 November 2020 to 16 November 2020, in five fix sites after a 10-day information campaign on T2DM. The SD CodeFreeTM glucose analyser was used to diagnose T2DM. Multivariable logistic regression was used to identify the associate factors.

**Results:**

A total of 1200 (95%) volunteered out of 1330 people were enrolled, which included 667 (52.27%) women. The mean age was 34.16 years (standard deviation: 12.42). Overall, 40.28% were abdominally obese and 31.43% hypertensive. The prevalence of T2DM was 10.74% (95% confidence interval [95% CI]: 9.15; 12.56). In multivariate analysis, being aged or greater than 35 years (adjusted odds ratio [ORa]: 2.30; 95% CI: 1.42; 3.72), having a family history of diabetes (ORa = 1.55; 95% CI: 1.006; 2.40), being overweight (ORa = 1.69; 95% CI: 1.09; 2.62), being obese (ORa = 1.80; 95% CI: 1.08; 3.00), being a known hypertensive (ORa = 2.92 95% CI: 1.64; 5.19) and having high blood pressure on the day of the survey (ORa = 1.86; 95% CI: 1.22; 2.85) increased significantly the probability to present T2DM.

**Conclusion:**

Community screening is useful to identify T2DM. A national programme to control diabetes mellitus and its associated risk factors is urgently needed in Burkina Faso.

**Contribution:**

This study will enable early detection of diabetes mellitus and its management in order to prevent or delay the onset of complications.

## Introduction

By 2050, over 1.31 billion people worldwide could be living with diabetes mellitus. The increase in the prevalence of diabetes mellitus is attributable to the rise in type 2 diabetes mellitus (T2DM).^1^ The main modifiable risk factors for T2DM are a sedentary lifestyle, a poor diet, obesity, smoking and excessive alcohol consumption.^2,3,4^ The most significant increase in the prevalence of diabetes mellitus is likely to occur in developing countries.^5,6^

In the African region of the World Health Organization (WHO), an estimated 24 million people were living with diabetes mellitus in 2021.^5^ In Burkina Faso (BF), the steps survey carried out in 2021 reported a national diabetes mellitus prevalence of 7.6% (95% confidence interval [CI]: 5.7; 9.5).^7^

The implementation of preventive measures, early diagnosis and effective treatment are essential for reducing the burden of diabetes mellitus^8^ contributing to the achievement of Sustainable Development Goal 3. However, the prevention and management of diabetes mellitus represent a major challenge for healthcare systems in sub-Saharan Africa, which is characterised by poverty, inadequate infrastructure and insufficient healthcare personnel to cope with the burden of diabetes mellitus.^9,10^ In addition, the region also faces the burden of communicable diseases. The majority of diabetics discover their disease through the cardinal signs of diabetes mellitus (27%) or at the onset of complications (61.53%).^10,11^ Screening for diabetes mellitus should be an entry point into the prevention and control of non-communicable diseases.^8^ The majority of diagnosed cases of diabetes mellitus are carried out by doctors in healthcare facilities, but people’s access to doctors remains very limited in sub-Saharan Africa and particularly in BF. In the WHO African region, 53.6% of diabetics were unaware of their condition.^12^ In Burkina Faso, 79% of participants in the steps 2021 survey in urban areas had never measured their blood glucose levels.^7^

Community screening could be an effective strategy for identifying people with undiagnosed type 2 diabetes. However, few data have been reported on the prevalence of undiagnosed diabetes mellitus in BF. The aim of this study was to determine the prevalence of undiagnosed diabetes mellitus and to investigate its associated factors in the urban population.

## Research methods and design

### Study framework

**FIGURE 1:**
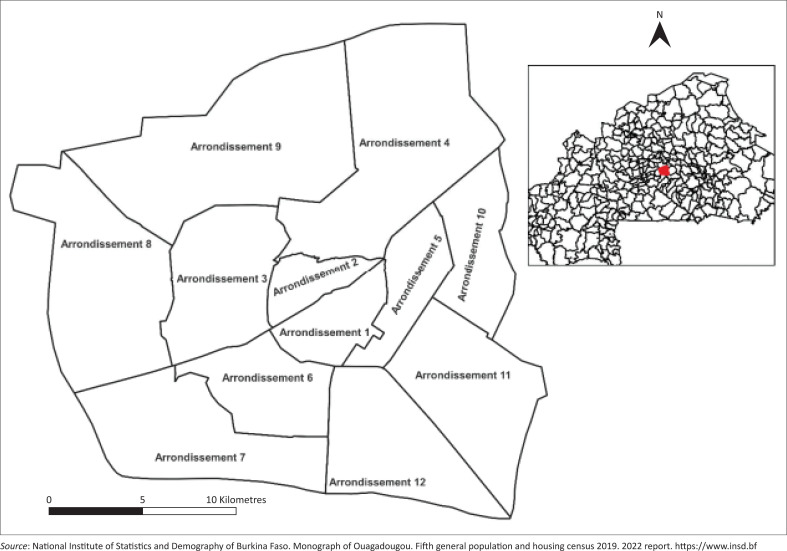
Map of the city of Ouagadougou with its arrondissements.

The study took place in the capital city of BF, Ouagadougou. The urban commune of Ouagadougou is located in the province of Kadiogo, in the Central region. Ouagadougou comprises 12 arrondissements and 55 sectors (Figure 1)^9^ and is the largest city. The commune has a population of 2 415 266, including 1 231 709 women and 1 183 557 men, living in 502 938 households. The majority of the population is very young, as is the country as a whole, with an average age of 23.8 years. Average population density is high in the commune, reaching over 4300 inhabitants per km^2^. The literacy rate for the population aged 15 and over is 61.2%. Civil servants represented 50.0%, 5.3% were unemployed and 44.7% were in the informal sector.^13^

### Type and study population

We conducted a community-based cross-sectional study from 11 November 2020 to 16 November 2020. The target population of the study was the adult population aged at least 18 years residing in the city of Ouagadougou.

### Sampling and data collection

A screening campaign was carried out in conjunction with World Diabetes Day 2020. A series of activities was initiated (public conferences, television and radio broadcasts on diabetes mellitus, popular cross-country events) from 11 November 2020 to 16 November 2020, including a diabetes mellitus screening campaign. Five arrondissements (numbers 1, 2, 3, 5 and 12) out of the 12 in the city of Ouagadougou were randomly selected and a fixed site was identified in each arrondissement. The survey was carried out during coronavirus disease 2019 (COVID-19) pandemic in BF. This study was part of a large study on diabetes mellitus during COVID-19 pandemic. The screening campaign was preceded by a 10-day media information campaign on television, radio and the web. Information was given in French and then translated into the two most widely spoken national languages (Mooré and Dioula).

All screening participants who were at least 18 years of age and had given oral consent were included in the study. Pregnant women and participants who declared that they were already taking drugs for diabetes were not included. Participants whose blood glucose results were not available were excluded from the study.

The data collection tool was an anonymous questionnaire. The SD CodeFree^TM^ glucose analyser, marketed by SD BIOSENSOR, Inc., was used to measure capillary blood glucose. The team of interviewers was made up of nurses and doctors trained in anthropometry, capillary glucose and blood pressure measurement and administering the questionnaire. The questionnaire was orally translated into Mooré or Dioula for those who did not understand French.

To establish the diagnosis of diabetes mellitus, capillary whole blood was taken by finger prick (a blood drop of about 1 mL) and immediately analysed using an SD CodeFree^TM^ glucose analyser. A control solution test was performed each time a new bottle of strips was opened. All other procedures described by the manufacturer were followed.

Blood pressure was measured with a mercury sphygmomanometer in the sitting position, using the right arm, after a rest period of at least 5 min. Three measurements were taken during the interview. The first reading was discarded, and the average of the last two readings was used for analysis. The measurement was taken on both arms to detect any difference in blood pressure between the arms. If this was the case, the higher blood pressure reading was used.

Height was measured in the standing position in centimetres (cm) using a measuring tape. Weight was measured with subjects in light clothing and without shoes, using a digital floor scale to the nearest 0.1 kg. Body mass index (BMI) was calculated by dividing weight (kg) by the square of height (m^2^). In the standing position, waist circumference was measured using a tape measure positioned midway between the lower ribs (below the last rib) and the iliac crest (the upper part of the pelvic bone). The value was measured to the nearest centimetre (cm) at the end of a normal exhalation at navel height.

### Study variables

#### Dependent variables

Diabetes mellitus was the dependent variable in our study. Capillary blood glucose was a continuous variable reported in mmol/L. Diabetes mellitus was defined as fasting capillary glucose (after at least 8 h of fasting) ≥ 6.1 mmol/L or postprandial glucose ≥ 11.1 mmol/L.^7,14^

A history of hyperglycaemia refers to the participant’s own declaration, regardless of the conditions under which the blood sample was taken or to the diagnostic criteria for diabetes.

#### Independent variables

Sex was categorised as female or male. Age was a discrete quantitative variable reported in years. The family history of diabetes mellitus was categorised into three classes: no family history, first-degree family history and second-degree family history. First-degree family history included close relatives: father, mother, children, sister and brother. Second-degree relatives included distant relatives such as grandparents, aunts, uncles and cousins. Body mass index is a continuous variable expressed in kg per m^2^. Normal weight, overweight and obesity were defined by BMI values between 18 kg/m^2^ and 24.9 kg/m^2^, between 25 kg/m^2^ and 29.9 kg/m^2^ and greater than or equal to 30 kg/m^2^. Waist circumference was a continuous variable whose value was expressed in centimetres (cm). Physical activity was coded into two classes: at least 30 min of daily physical activity or not. Daily consumption of fruit and vegetables was coded into two classes: daily versus not. For vegetables, the aim was to ascertain respondents’ daily consumption of the equivalent of one bowl of fresh, raw, leafy green vegetables (spinach, salad, etc.), half a bowl of other vegetables, cooked or raw, cut into small pieces (tomatoes, squash, green beans, etc.) or half a bowl of vegetable juice. In the case of fruit, the aim was to ascertain the daily consumption frequency of the equivalent of a medium-sized fruit (orange, banana, apple, etc.) or half a glass of fruit in pieces, cooked or in syrup or half a glass of fruit juice (without artificial flavouring). Hyperglycaemia was dichotomised (yes or no). The use of anti-hypertensive treatment was dichotomised (yes or no). Arterial hypertension was defined as systolic blood pressure ≥ 140 mmHg and/or diastolic blood pressure ≥ 90 mmHg.

### Data quality assurance

All the participants had unique identifier and names were not recorded. Outliers for the dependent variable: the glycaemia and anthropometric variables (weight, length) were excluded from the analysis.

### Analysis

The study population was described in terms of frequencies and percentages for qualitative variables. Quantitative variables were described by their mean and standard deviation. We estimated the prevalence of diabetes mellitus and its 95% CI. Factors associated with diabetes were identified by logistic regression. Variables associated in univariable analysis at the 20% threshold were included in a multivariable model. The final model was obtained using a step-by-step manual step-down procedure. The significance level in the final model was set at 5%. Analyses were carried out using Stata 14 (Stata Corp LLC).

### Ethical considerations

Authorisation from the administrative and health authorities of the Central region was obtained for this study. Ethical clearance to conduct this study was obtained from the National Ethic Committee of Burkina Faso (Comité d’Ethique pour la Recherche en Santé) (No. 2020-8-146).

It was a secondary objective of this study. The oral consent of the participants to the screening was a prerequisite to the administration of the questionnaire, which was anonymous.

## Results

A total of 1276 individuals took part in the study, including 667 (52.27%) women. The mean age was 34.16 years (standard deviation: 12.42 years), with extremes ranging from 18 years to 80 years. Two-thirds of participants (78.84%) were under 45 years old. In our study population, 40.28% were abdominally obese, 26.49% were overweight and 14.81% were obese. The majority of participants reported no family history of diabetes. Half of the participants in our study performed at least 30 min of daily physical activity, with walking being the most commonly reported activity. Less than one in five participants (16%) consumed fruit and vegetables on a daily basis. The prevalence of hypertension was 31.43% (Table 1).

**TABLE 1:** Sociodemographic, anthropometric, lifestyle and terrain characteristics of participants (*N* = 1276).

Variables	Number (*n*)	Proportion (%)
**Gender**	1276	-
Female	667	52.27
Male	609	47.13
**Age range (years)**	1276	-
< 35	747	58.54
35–45	259	20.30
45–55	164	12.85
55–64	77	6.03
> 64	29	2.27
Public sector (civil servants)	435	34.66
Student	414	32.99
Self-employed (liberal)	188	14.98
Private sector	131	10.44
Unemployed	87	6.85
**Marital status**	1257	-
Couple	654	52.03
Single	568	45.19
Widower	30	2.39
Divorced	5	0.40
**Mode of travel**	1263	-
Motorcycle	810	64.13
Moped	202	15.99
Pedestrian	166	13.14
Car	85	6.73
**Meal location**	1258	-
At home	995	79.09
Out of the house	263	20.91
**Family history of diabetes†**	1276	-
No previous history	1027	80.49
1st degree family history‡	132	10.34
2nd degree family history§	117	9.17
**30 min of daily physical activity**	1276	-
Yes	631	49.45
No	645	50.55
**Type of physical activity**	1261	-
Walk	571	45.28
Work	315	24.38
Leisure	238	18.87
No	137	10.86
**Daily consumption of fruit and vegetables**	1276	-
Not every day	1077	84.40
Every day	199	15.60
**Anti-hypertensive drugs**	1276	-
Yes	94	7.37
No	1182	92.63
**History of hyperglycaemia**	1276	-
Yes	60	4.70
No	1216	95.30
**Waist circumference in cm**	1276	-
No abdominal obesity¶	762	59.72
Presence of abdominal obesity#	514	40.28
**BMI (kg/m^2^)**	1276	-
Normal††	749	58.70
Overweight‡‡	338	26.49
Obesity§§	189	14.81
**Arterial hypertension**	1276	-
Normal blood pressure	875	68.57
High blood pressure on the day of the survey	307	24.06
Being a known hypertensive	94	7.37
**Contraceptive**	632	-
Yes	187	29.59
No	445	70.41

BMI, body mass index.

† , Past history;

‡ , 1st Degree: close relatives, for example father, mother, children, sister, brother;

§ , 2nd, Degree: distant relatives, for example grandparents, aunts, uncles, cousins;

¶ , No abdominal obesity: waist circumference < 80 cm for women and < 94 cm for men; #, Presence of abdominal obesity: waist circumference ≥ 80 cm for women and ≥ 94 cm for men;

†† , Normal: BMI between 18 kg/m^2^ – 25 kg/m^2^;

‡‡ , Overweight: BMI between 25 kg/m^2^ – 30 kg/m^2^;

§§ , Obesity: BMI ≥ 30 kg/m^2^.

The prevalence of diabetes was 10.74% (95% CI: 9.15; 12.56). In univariate analysis, several variables were associated with diabetes. These included male gender (crude odds ratio [ORb]: 0.61; 95% CI: 0.42; 0.88), age at least 35 years (ORb: 3.07; 95% CI: 1.95; 4.84), anti-hypertensive medication (ORb: 3.46; 95% CI: 2.10; 5.69), history of hyperglycaemia (ORb: 2.70; 95% CI: 1.44; 5.06), abdominal obesity (ORb: 3.11; 95% CI: 2.14; 4.51), being overweight (ORb: 2.65; 95% CI: 1.76; 3.99) or obesity (ORb: 2.95; 95% CI: 1.84; 4.74), living with a partner (ORb: 3.35; 95% CI: 2.18; 5.15), personal history of hypertension (ORb: 4.83; 95% CI: 2.85; 8.18) and elevated blood pressure on the day of the survey (ORb: 2.65; 95% CI: 1.78; 3.95) significantly increased the probability of developing diabetes. While the practice of physical activity (ORb: 0.45; 95% CI: 0.31; 0.66), being student (ORb: 0.58; 95% CI: 0.34; 0.98), eating away from home (ORb: 0.56; 95% CI: 0.38; 0.83) and riding a motorcycle (ORb: 0.52; 95% CI: 0.27; 0.92) significantly reduced the probability of developing diabetes (Table 2).

**TABLE 2:** Univariate and multivariate analyses.

Variables	Total diabetic		Univariate			Multivariate		
	*n*	*N*	ORb	95% CI	*p*	ORa	95% CI	*p*
**Gender**	-	-	-	-	-	-	-	-
Female	86	667	1.000	-	-	-	-	-
Male	51	609	0.061	0.42; 0.88	0.010	-	-	-
**Age range (years)**	-	-	< 0.001	-	-	-	-	0.010
< 35	43	747	1.000	-	-	1.00	-	-
35–45	41	259	3.007	1.95; 4.84	< 0.001	2.30	1.042; 3.72	0.001
45–55	30	164	3.066	2.21; 6.05	< 0.001	2.18	1.025; 3.79	0.006
55–64	17	77	4.063	2.49; 8.62	< 0.001	2.96	1.052; 5.73	0.001
> 64	6	29	4.027	1.65; 11.04	0.003	2.68	0.098; 7.32	0.050
**Family history of diabetes†**	-	-	-	-	-	-	-	-
Yes	35	249	1.048	0.98; 2.23	0.060	1.55	1.006; 2.40	0.040
No	102	1027	1.000	-	-	1.00	-	-
**30 min of daily physical activity**	-	-	-	-	-	-	-	-
Yes	46	645	0.045	0.31; 0.66	< 0.001	-	-	-
No	91	631	1.000	-	-	-	-	-
**Daily consumption of fruit and vegetables**	-	-	-	-	-	-	-	-
Not every day	122	1077	1.000	-	-	-	-	-
Every day	15	199	0.063	0.36; 1.11	0.011	-	-	-
**Anti-hypertensive drugs**	-	-	-	-	-	-	-	-
Yes	25	94	3.046	2.10; 5.69	< 0.001	-	-	-
No	112	1182	1.000	-	-	-	-	-
**History of hyperglycaemia**	-	-	-	-	-	-	-	-
Yes	14	60	2.070	1.44; 5.06	0.002	-	-	-
No	123	1216	1	-	-	-	-	-
**Waist circumference (cm)**	-	-	-	-	-	-	-	-
No abdominal obesity‡	48	762	1.000	-	-	-	-	-
Presence of abdominal obesity§	89	514	3.011	2.14; 4.51	< 0.001	-	-	-
**BMI (kg/m^2^)**	-	-	-	-	-	-	-	0.004
Normal¶	50	749	1.000	-	-	-	-	-
Overweight††	54	338	2.065	1.76; 3.99	< 0.001	1.69	1.009; 2.62	0.010
Obesity‡‡	33	189	2.095	1.84; 4.74	< 0.001	1.80	1.008; 3.00	0.020
**Marital status**	-	-	-	-	< 0.001	-	-	-
Only	29	568	1.000	-	-	-	-	-
Widower	4	30	2.085	0.93; 8.73	0.060	-	-	-
Couple	100	654	3.035	2.18; 5.15	< 0.001	-	-	-
**Profession**	1255	-	-	-	0.001	-	-	-
Public (civil servants)	40	435	1.000	-	-	-	-	-
Unemployed	17	87	2.039	1.28; 4.46	0.006	-	-	-
Student	23	414	0.058	0.34; 0.98	0.045	-	-	-
Private	19	131	1.067	0.93; 3.01	0.084	-	-	-
Self-employed (liberal)	36	188	2.033	1.43; 3.88	0.001	-	-	-
**Meal location**	-	-	-	-	-	-	-	-
At home	41	263	1.00	-	-	-	-	-
Out of the house	94	995	0.56	0.38; 0.83	0.005	-	-	-
**Type of physical activity**	-	-	-	-	0.001	-	-	-
No	27	137	1.00	-	-	-	-	-
Walk	67	571	0.54	0.33; 0.88	0.015	-	-	-
Leisure	15	238	0.27	0.14; 0.53	< 0.001	-	-	-
Work	27	315	0.38	0.21; 0.68	0.001	-	-	-
**Means of transport**	-	-	-	-	0.056	-	-	-
Feet	17	166	0.53	0.25; 1.12	0.010	-	-	-
Moped	24	202	0.62	0.31; 1.26	0.019	-	-	-
Motorcycle	79	810	0.52	0.27; 0.92	0.002	-	-	-
Car	15	85	-	-	-	-	-	-
**Hypertension (BP taken on the day of collection)**	-	-	-	-	< 0.001	-	-	< 0.001
Normal blood pressure	61	875	1.00	-	-	1.00	-	-
High blood pressure on the day of the survey	51	307	2.65	1.78; 3.95	< 0.001	1.86	1.22; 2.85	0.004
Being a known hypertensive	25	94	4.83	2.85; 8.18	< 0.001	2.92	1.64; 5.19	< 0.001

ORb, crude odds ratio; ORa: adjusted odds ratio; CI, confidence interval; BP, blood pressure.

† , Past history;

‡ , No abdominal obesity: Waist circumference < 80 cm for women and < 94 cm for men;

§ , Presence of abdominal obesity: waist circumference ≥ 80 cm for women and ≥ 94 cm for men;

¶ , Normal: BMI between 18 kg/m^2^ – 25 kg/m^2^;

†† , Overweight: BMI between 25 kg/m^2^ and 30 kg/m^2^;

‡‡ , Obesity: BMI ≥ 30 kg/m^2^.

In multivariate analysis, being aged greater than 35 years (adjusted odds ratio [ORa]: 2.30; 95% CI: 1.42; 3.72), having a family history of diabetes (Ora: 1.55; 95% CI: 1.006; 2.40), being overweight (ORa: 1.69; 95% CI: 1.09; 2.62) or obesity (Ora: 1.80; 95% CI: 1.08; 3.00), having a personal history of hypertension (Ora: 2.92; 95% CI: 1.64; 5.19) and having elevated blood pressure on the day of the survey (Ora: 1.86; 95% CI: 1.22; 2.85) significantly increased the probability of presenting with diabetes (Table 2).

## Discussion

This study estimated the prevalence of undiagnosed diabetes mellitus and identified its associated factors in an urban setting during a mass screening campaign in BF. One patient in 10 was diabetic and unaware of their condition. An age of at least 35 years and a personal history of hypertension and elevated blood pressure on the day of the survey were factors significantly associated with diabetes mellitus in our study participants.

The prevalence of undiagnosed diabetes mellitus in urban areas was 10.74% in our study. These identified cases of diabetes were referred for medical management. In BF, diabetes mellitus prevalence studies reported proportions ranging from 4.1% to 12.4%.^14,15,16,17^ A systematic review reported that the mean prevalence of undiagnosed diabetes mellitus among adults in Africa was 3.85% (95% CI: 3.10; 4.60), with geographical differences including 4.43% (95% CI: 3.12; 5.74) in East Africa; 4.72% (95% CI: 2.64; 6.80) in West Africa; 4.27% (95% CI: 1.77; 6.76) in North Africa and 1.46% (95% CI: 0.57; 2.34) in Southern Africa.^18^ Another systematic review coupled with a meta-analysis found an overall prevalence of undiagnosed diabetes mellitus in the African population of 5.37% (95% CI: 4.57; 6.81).^19^ The same review indicated that the prevalence of undiagnosed diabetes mellitus in the urban population (8.68%, 95% CI: 5.33; 12.03) was twice as high as in the rural population (3.93%, 95% CI: 2.91; 4.95).^19^ In Ethiopia, a systematic review coupled with a meta-analysis found that the prevalence of undiagnosed diabetes mellitus was 5.75%, 95% CI: 3.90; 7.59%.^20^ Recent studies in the same country (Ethiopia) found prevalences of 4.5% (95% CI: 2.9; 7.4) in urban areas,^21^ 8.64% (95% CI: 6.7; 11.2)^22^ and 14.7% (95% CI: 11.1; 18.3) in socially marginalised communities.^23^ The high prevalence reported in our study reflects the increase in diabetes mellitus in sub-Saharan Africa. Differences between studies may reflect contextual differences in lifestyle and dietary habits between countries and regions.^24^ The high prevalence of diabetes mellitus found in our study could also be explained by the awareness campaign conducted in the media for at least the previous 10 days. Thus, subjects with at least one cardiovascular risk factor are more sensitive to messages calling for screening for other risk factors such as diabetes mellitus. These may have been prompted by risk factors such as heredity, overweight or obesity or high blood pressure. This may lead to an overestimation of the prevalence of diabetes mellitus.^15,17,25,26^ Such screening provides an opportunity for volunteers with risk factors to be monitored and treated at an early stage, thereby helping to prevent cardiovascular events and reduce mortality.^8,27^ According to data from the International Diabetes Federation (IDF), 53.6% of diabetes mellitus cases are undiagnosed in sub-Saharan Africa.^12^

In our study, only 4.7% of volunteers reported a history of hyperglycaemia, in the context of low socio-economic status and a lack of health coverage. In fact, 78.7% of participants in the steps 2021 survey in urban areas had never had their blood glucose levels measured.^7^ Millogo et al. in BF found that 75.3% were measuring blood glucose for the first time.^16^ Ultimately, this type of mobile screening would make it possible to identify patients at risk of developing diabetes mellitus and to consider confirmation by measurement of venous glycaemia under the recommended conditions.

An age of at least 35 years was significantly associated with diabetes mellitus in our study. Several authors have reported in urban studies that advanced age is associated with a greater risk of diabetes mellitus.^16,21,24,25,28,29^ Indeed, Millogo et al. in BF found that, compared with 25–34 year-olds, belonging to the 45–54 years and 55–64 years age groups increased the risk of developing diabetes mellitus by a factor of 1.5 and 1.9, respectively.^24^ Age ≥ 45 years was associated with the occurrence of diabetes mellitus in workers.^17^ Wierusz-Wysocka et al. concluded in their study, including professionally active city dwellers, that screening for diabetes mellitus should be carried out mainly in subjects over 35 years of age, as is the case in our volunteers.^30^ In a multicentre study, age over 50 years was associated with diabetes mellitus diagnosis (OR: 3.3; 95% CI: 1.4; 7.8).^31^ The same observation was made in rural areas, as shown by Séré et al. in BF^26^ and Diawara et al. in Mali.^32^ In 2021, the American Diabetes Association (ADA) recommended diabetes mellitus screening for all subjects aged 45 years and over, as well as for subjects with multiple risk factors for diabetes mellitus.^14^ Contrary to the results of these studies, Eurasmus et al. in South Africa (> 40 years of age)^33^ and Yaméogo et al. in BF (> 35 years)^15^ found that age made no statistically significant difference to the occurrence of diabetes mellitus in their study populations.

Nevertheless, the association between diabetes and age is consistent with the data and demonstrated that the proportion of diabetics in each age group should increase as the population ages. Diabetes was present in 20.69% (*n* = 6/29) of subjects over 64 years in our study. This high incidence may be explained in part by increased life expectancy and sedentary lifestyles in developing countries. Age and ethnicity are the main non-modifiable factors associated with diabetes mellitus in Africa.^34^

In our study, a family history of diabetes was a variable significantly associated with diabetes mellitus (ORa: 1.55; 95% CI: 1.006; 2.40). Issa et al. in BF reported that a family history of diabetes mellitus was associated with diabetes.^17^ Several studies also concluded that a family history of diabetes mellitus increased the risk of developing diabetes mellitus.^19,20,21,22,34,35,36,37^ The results of a multicentre study found that family history of diabetes mellitus increased the risk of developing diabetes by twofold.^31^ Diawara et al. even in rural Mali, found that only family history of diabetes mellitus was independently associated with a more than 14-fold risk of developing diabetes mellitus.^32^ Indeed, the knowledge that one is at greater risk of developing diabetes mellitus may encourage relatives of patients concerned to adopt a healthier lifestyle.

In multivariate analysis, the following anthropometric factors were associated with diabetes mellitus: obesity (ORa: 1.80; 95% CI: 1.08; 3.00) and overweight (ORa: 1.69; 95% CI: 1.09; 2.62). In BF studies, overweight and/or obesity were independently associated with the onset of diabetes mellitus.^16,24,25,28,29^ This association was reported even in rural areas by Séré et al. in BF.^26^ A meta-analysis conducted in 49 developing countries found that people suffering from malnutrition, overweight or obesity were more exposed to diabetes.^38^ Mayega et al. in Uganda^36^ Duboz et al. in Senegal^2^ and Damtie et al. in Ethiopia^21^ also showed that overweight and/or obesity were risk factors for diabetes mellitus. Wierusz-Wysocka et al. in Poland concluded in their study of professionally active city dwellers that diabetes mellitus screening should be carried out mainly in overweight or obese subjects.^30^ The same observation was made by Eurasmus et al. in South Africa, who found among workers an association between overweight and/or obesity and diabetes mellitus.^33^

A study from BF showed that the prevalence of diabetes also increased with abdominal obesity.^16^ Abdominal obesity was a factor associated with diabetes in several studies.^2,3,39,40,41^ Diawara et al. made the same finding in their study carried out in rural Mali (*p* = 0.013).^32^ A systematic review concluded that BMI (overweight/obesity and abdominal obesity) would be a major predictive factor for diabetes propensity up to 2050.^4^

A high blood pressure on the day of the survey increased the probability of diabetes in our study. Hypertension has also been reported as a factor associated with diabetes mellitus in previous studies in BF.^28,29^ Furthermore, a history of hypertension was independently associated with a risk of developing diabetes mellitus in a review of the literature.^21,31^ Diawara et al. concluded that hypertension was associated with the onset of diabetes mellitus in rural Mali.^32^ Our study argues in favour of active screening and awareness-raising for hypertension in the population, as preventing hypertension would prevent diabetes mellitus and vice versa.

### Limitation of the study

The main limitation of this study is inherent in its cross-sectional design, which makes it impossible to draw causality. In addition, the study data were collected data at fixed sites, including volunteers. This can later lead to biased selection. Blood glucose measurements were taken on a single occasion and could not be repeated, as required for the diagnosis of diabetes mellitus in the standards of excellence,^42^ and the device used measured total blood glucose instead of plasma glucose, widely used in recent studies to cause overestimation bias. The measurement of most exposures was based on participant declarations. This can lead to information bias. Some well-known metabolic risk factors for diabetes mellitus were not included in the study, such as lipids and a history of foetal macrosomia.

## Conclusion

Community screening is useful for African populations in the absence of health insurance. It could be an effective strategy for identifying people with undiagnosed type 2 diabetes in low-incomes countries, such as BF. It allows identifying individuals at risk and people living with diabetes mellitus who was unaware of their condition. Our study highlights the need to set up an integrated national programme to control diabetes mellitus and other associated cardiovascular risk factors through periodic data collection, information and awareness-raising in BF.
